# Unveiling the mystery of prolonged fever with lymphadenopathy and skin rashes– Kikuchi-Fujimoto disease with discoid lupus erythematosus: A case report

**DOI:** 10.1016/j.jdcr.2026.07.030

**Published:** 2026-08-22

**Authors:** Naima Luqman, Mahym Mansoor, Humna Khalid, Mohsin Sajjad, Arooba Iftikhar, Maryam Tariq, Komal Naseer

**Affiliations:** Department of Dermatology, Bahawal Victoria Hospital, Quaid-e-Azam Medical College, Bahawalpur, Pakistan

**Keywords:** discoid lupus erythematosus, interface dermatitis, Kikuchi-Fujimoto necrotizing lymphadenitis, systemic lupus erythematosus

## Clinical findings

An 18-year-old woman presented with an 8-month history of multiple erythematous, well-demarcated, scaly plaques involving the right side of the scalp (Fig 1, *A*), occipital region, and right side of the face. The lesions were associated with scaling, hypopigmentation, and progressive alopecia. Three months before presentation, she developed intermittent low-grade fever, generalized fatigue, and bilateral cervical lymphadenopathy. There was no history of photosensitivity, oral ulcers, joint pain, Raynaud phenomenon, or weight loss.

**Fig 1:**
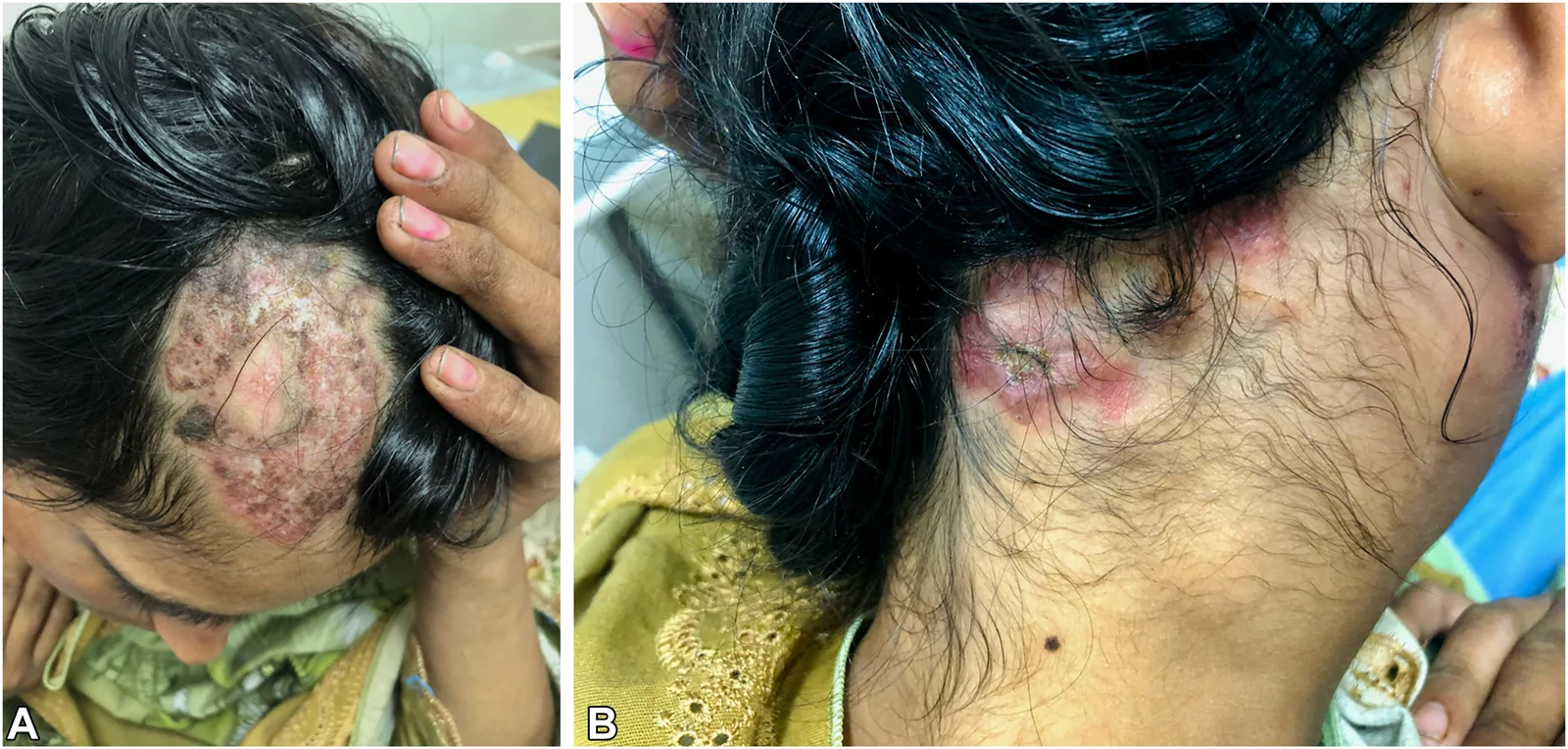
**A,** Scaly erythematous plaque on the right side of the scalp. **B,** Scaly erythematous plaques on the posterior neck with visible lymph nodes.

Cervical lymph nodes were enlarged, discrete, mobile, and mildly tender (Fig 1, *B*). Routine laboratory investigation findings were within normal limits, and antinuclear antibody and extractable nuclear antigen panel results were negative.

## Histopathologic findings

Histopathologic examination of the scalp lesion (Figs 2, *A, B*, and 3) revealed hyperkeratosis, follicular plugging, thickened basement membranes, interface lymphocytic dermatitis, and dermal mucin deposition. Cervical lymph node biopsy (Fig, 4) revealed necrotizing lymphadenitis with karyorrhectic debris and histiocytic infiltration.

**Fig 2:**
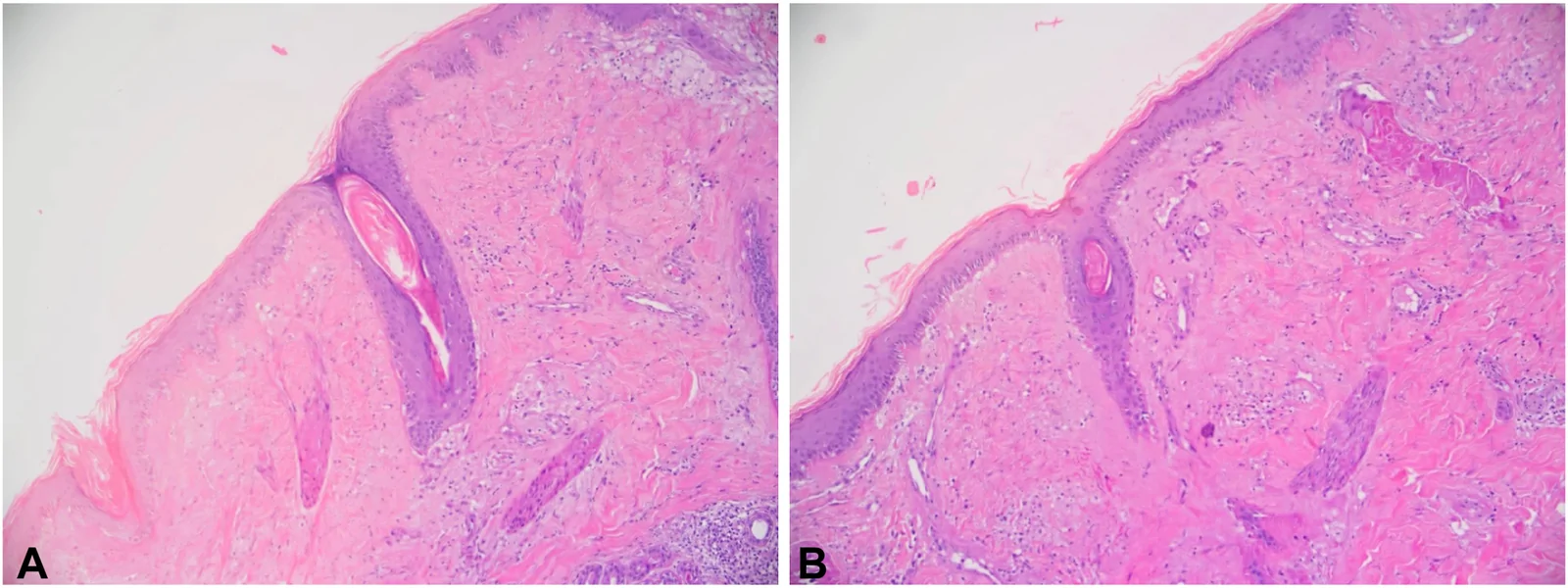
**A, B,** Hyperkeratosis, epidermal flattening with marked basal vacuolar damage; the underlying basement membrane is thickened.

**Fig 3:**
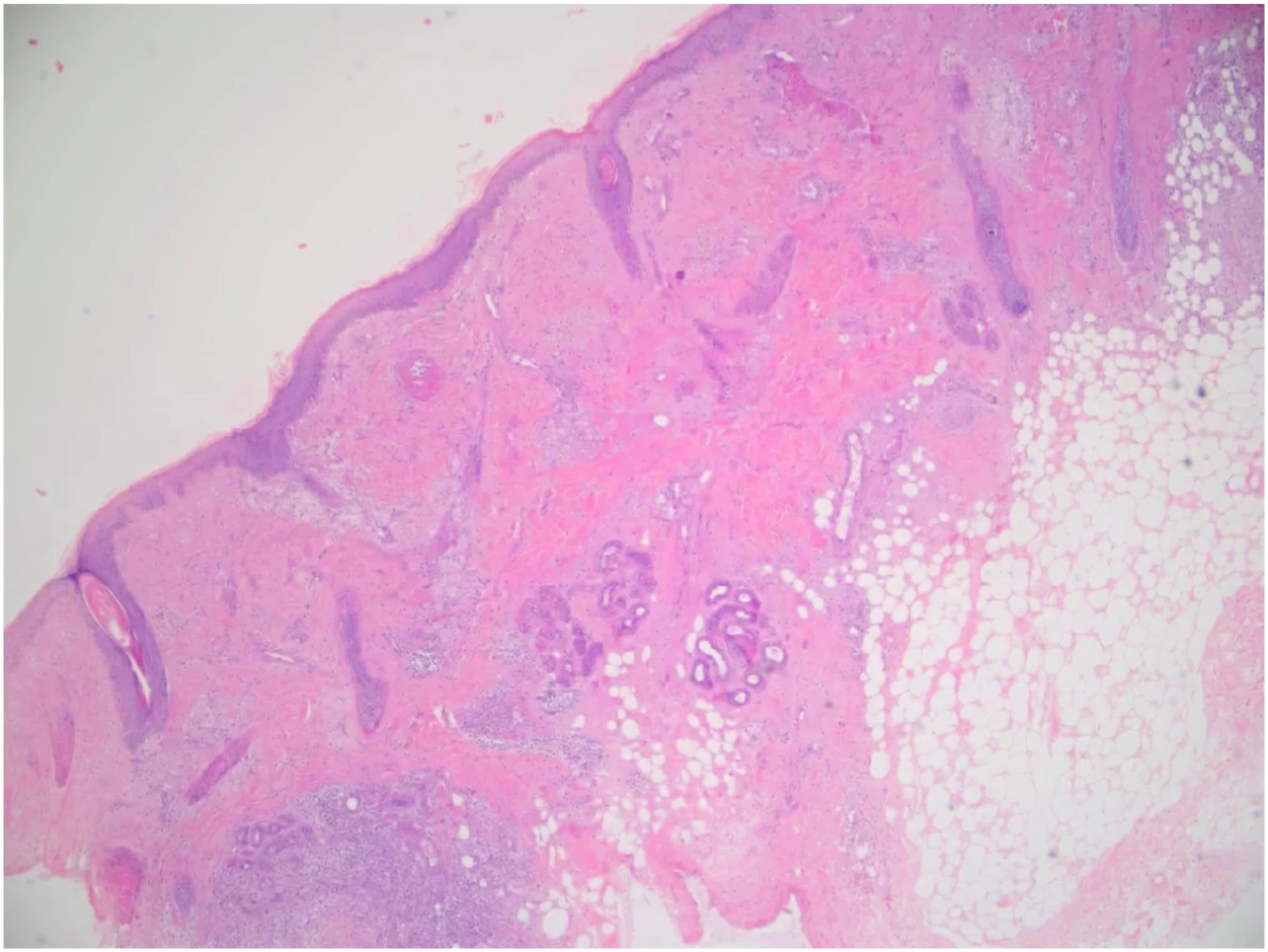
Follicular plugging, marked hyalinization, lympho-histiocytic infiltrate around vessels and adnexa extending into adipose tissue.

**Fig 4:**
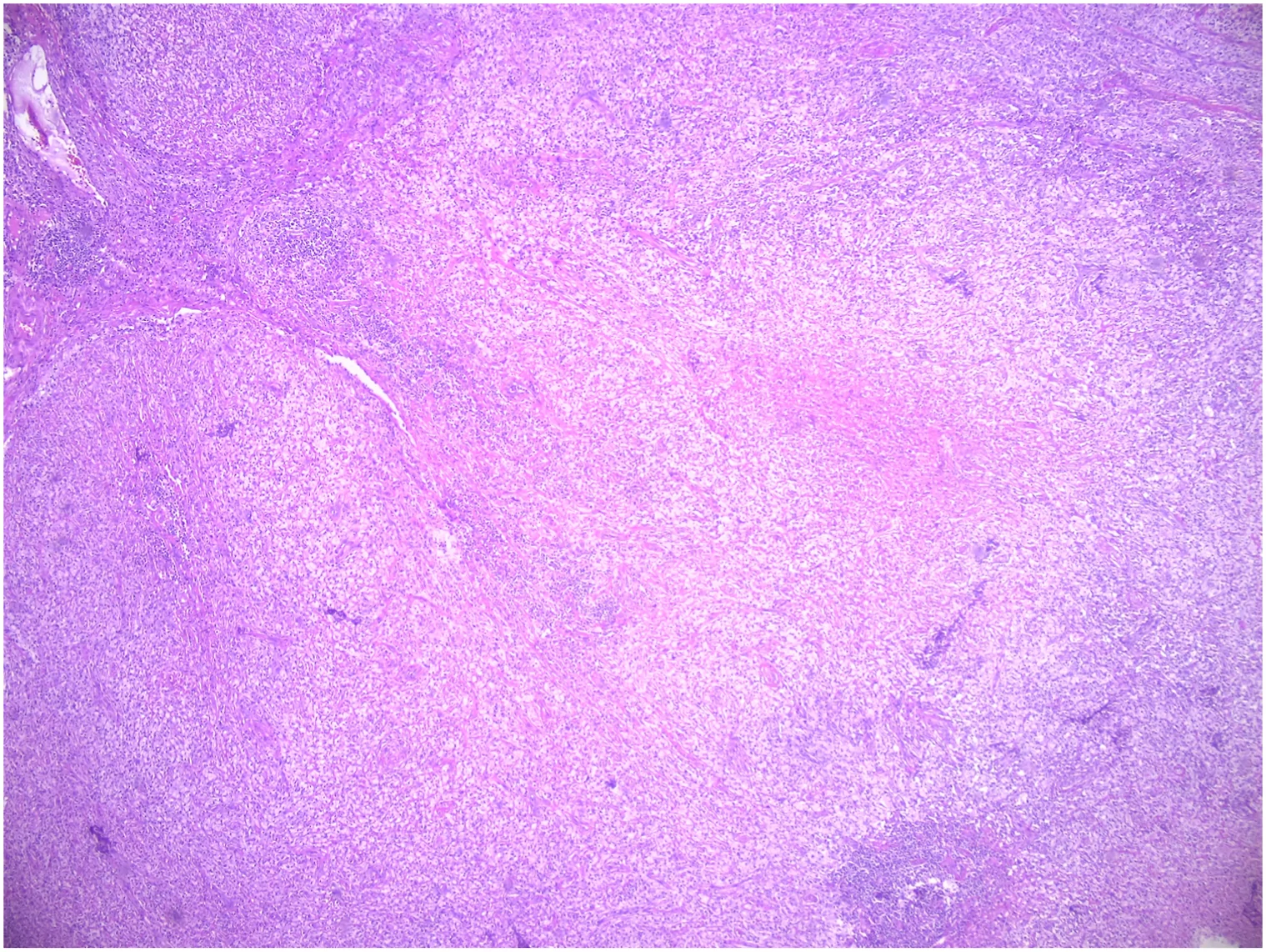
Lymph node with effaced architecture*;* the paler center shows areas of infarction with lymphocytes and plasma cells, and histiocytes are seen densely.

**Question 1: Which of the following is the most likely diagnosis?**

**A.** Sarcoidosis.

**B.** Lichen planopilaris.

**C.** Kikuchi-Fujimoto disease associated with discoid lupus erythematosus.

**D.** Non-Hodgkin's lymphoma.

**E.** Systemic lupus erythematosus.

**Answers:**

**A.** Sarcoidosis—Incorrect.

**B.** Lichen planopilaris—Incorrect.

**C.** Kikuchi-Fujimoto disease associated with discoid lupus erythematosus—Correct.

**D.** Non-Hodgkin's lymphoma—Incorrect.

**E.** Systemic lupus erythematosus—Incorrect.

## Discussion

Kikuchi-Fujimoto necrotizing lymphadenitis (KFNL) was first reported by Kikuchi and Fujimoto et al in 1972 in Japan. It is a rare, self-limited inflammatory disorder that typically affects young adults and is characterized by fever, constitutional symptoms, and cervical lymphadenopathy. KFNL is often misdiagnosed because of its clinical and histopathologic overlap with diseases such as systemic lupus erythematosus (SLE), lymphoma, herpes virus infections, and infectious mononucleosis. Importantly, progression to SLE has been reported in about 13% to 25% of patients with KFNL.^1^ This patient possessed several factors that are recognized predictors for the future development of SLE, including female gender, decreased cell count on complete blood examination, and cutaneous lesions showing interface dermatitis.^2^ These features place the patient at a higher risk for progression to systemic disease.

Patients with KFNL should be counseled regarding potential environmental and lifestyle triggers that may pave the way for the development of SLE, including excessive sunlight exposure, smoking, alcohol consumption, and the use of oral contraceptive pills. Preventive counseling forms an important component of long-term management in such patients. Hydroxychloroquine was initiated in this patient, as it delays progression to SLE in patients with KFNL and lupus spectrum disease.^3^

Hemophagocytic lymphohistiocytosis is a serious complication of KFNL due to a cytokine storm; there is hyperactivation of macrophages, natural killer cells, and cytotoxic T lymphocytes. Affected patients usually present with sepsis, bleeding, multiorgan failure, encephalopathy, and coma.^4^

Macrophage activation syndrome is another life-threatening complication of KFNL and must be identified early. Laboratory findings such as increased lactate dehydrogenase and lymphopenia may aid in diagnosis. Approximately 25.9% of patients with KFNL may develop macrophage activation syndrome, underscoring the importance of close monitoring.^5^ Although KFNL is a self-limiting condition, with complete recovery occurring in 1 to 6 months, recurrence has been reported in 3% to 4% of the patients. Management is largely supportive and includes analgesics, antipyretics, and nonsteroidal anti-inflammatory drugs tailored to symptom severity.

## Conflicts of interest

None disclosed.
